# Quadruple Burden of HIV/AIDS, Tuberculosis, Chronic Intestinal Parasitoses, and Multiple Micronutrient Deficiency in Ethiopia: A Summary of Available Findings

**DOI:** 10.1155/2015/598605

**Published:** 2015-02-12

**Authors:** Bemnet Amare, Beyene Moges, Andargachew Mulu, Sisay Yifru, Afework Kassu

**Affiliations:** ^1^Department of Medical Biochemistry, College of Medicine and Health Sciences, University of Gondar, Gondar, Ethiopia; ^2^Department of Microbiology, Immunology and Parasitology, College of Medicine and Health Sciences, University of Gondar, Gondar, Ethiopia; ^3^Department of Pediatrics, College of Medicine and Health Sciences, University of Gondar, Gondar, Ethiopia; ^4^Department of Microbiology, Immunology and Parasitology, Addis Ababa University, Addis Ababa, Ethiopia

## Abstract

Human immunodeficiency virus (HIV), tuberculosis (TB), and helminthic infections are among the commonest public health problems in the sub-Saharan African countries like Ethiopia. Multiple micronutrient deficiencies also known as the “hidden hunger” are common in people living in these countries either playing a role in their pathogenesis or as consequences. This results in a vicious cycle of multiple micronutrient deficiencies and infection/disease progression. As infection is profoundly associated with nutritional status resulting from decreased nutrient intake, decreased nutrient absorption, and nutrient losses, micronutrient deficiencies affect immune system and impact infection and diseases progression. As a result, micronutrients, immunity, and infection are interrelated. The goal of this review is therefore to provide a summary of available findings regarding the “quadruple burden trouble” of HIV, TB, intestinal parasitic infections, and multiple micronutrient deficiencies to describe immune-modulating effects related to disorders.

## 1. Introduction

Human immunodeficiency virus (HIV), tuberculosis (TB), and helminthic infections are among the commonest public health problems in the sub-Saharan African countries. Micronutrient deficiencies are an additional burden for these groups of population either playing a role in their pathogenesisor as a consequence ending up in a vicious cycle.

It is estimated that one-third of the world's population is latently infected with* Mycobacterium tuberculosis (M. tb)* and that each year about three million people die of TB [1, 2]. The emergence of drug-resistant strains is further worsening the threat [1]. Despite global research efforts, mechanisms underlying pathogenesis, virulence, and persistence of* M. tb* infection remain poorly understood [2]. In 1993, the World Health Organization (WHO) declared TB a global public health emergency [2]. The WHO global reports on TB showed that Ethiopia is among the top ten high burden countries in terms of prevalence or incidence cases of TB [1, 3]. Tuberculosis is the second leading cause of death from an infectious disease worldwide, only second to HIV. The HIV/AIDS pandemic, on the other hand, has had its most profound impact to date in sub-Saharan Africa. The majority of people living with HIV/AIDS (67%), new HIV infections (70%), and AIDS-related deaths (75%) are in this region, which only accounts for about 11-12% of the world's population [4]. With a national adult HIV prevalence of 2.1%, Ethiopia is one of the sub-Saharan countries most severely hit by the epidemic. The dominant mode of transmission of the virus among adults is heterosexual transmission while mother-to-child transmission accounts for more than 90% of pediatric HIV infections [5, 6].

About three billions of people are infected with one or more species of intestinal parasites which are distributed virtually throughout the world, with higher prevalence rates in many tropical and subtropical regions [7, 8]. These parasites release multitude of antigens into the circulation which would lead to chronic activation of the immune system [9–11]. In sub-Saharan Africa, where the prevalence of parasitic infections is very high, a dominant type-2 T helper polarized immune response has been reported [12] and suggested to increase susceptibility to both* M. tb* and HIV. Coinfection also hastens progression of their respective diseases [13–15].

Along with these infections, single or multiple micronutrient deficiencies have been shown to influence host resistance mechanisms, thus altering the susceptibility to infectious diseases [9–15]. Knowledge of the immune-modulating effects of micronutrients and their interactions with HIV, TB, and chronic intestinal parasitic infection which cause major public health problem in Ethiopia (Table 1), is of great importance in planning comprehensive strategies to promote health through nutrition and to augment specific therapy. The goal of this review is to provide a summary of available findings and summarize current state of knowledge regarding the “quadruple burden,” multiple micronutrient deficiency, HIV, TB, and intestinal parasitic infection, and to describe immune-modulating effects of these disorders.

## 2. Methods

This review was on paper after reviewing the relevant information available about the burden of HIV, TB, intestinal helminthes, and micronutrient deficiencies in Ethiopia and current evidences on their interactions from Hinari (http://www.who.int/hinari/en/) and PubMed (http://www.ncbi.nlm.nih.gov/pubmed). Although much has been published in the last 10 years regarding our topic, we still need more information so as to understand the issues that will help us develop effective programs in Ethiopia and other African countries with similar conditions. Therefore, we have also used more literatures which are less than ten years old.

## 3. Interactions between Micronutrient Deficiency and Infection 

Micronutrients, immunity, and infections are interrelated [22]. Undernourished persons show immune dysfunction, which predisposes them to infections [23, 24]. Micronutrient deficiencies, also known as “hidden hunger,” disturb the normal function of the immune system components, weakening immune defenses, and increasing susceptibility to various infectious diseases [24–29]. Infection, in turn, is associated with profound effects on nutritional status resulting from decreased nutrient intake due to loss of appetite, decreased nutrient absorption as a result of intestinal damage and malabsorption, and nutrient losses arising from diarrhea and increased urinary excretion. A number of micronutrient deficiencies have been reported in persons with TB [16, 17, 30–36] and HIV infection [21, 37–44] and among those with intestinal parasitic infections [18, 19, 45–49]. The risk of multiple micronutrient deficiencies is high in developing countries, due to monotonous diets based on staple foods of low nutrient density [50].

## 4. Factors Contributing to Micronutrient Deficiencies during Infections 

**Figure 1:**
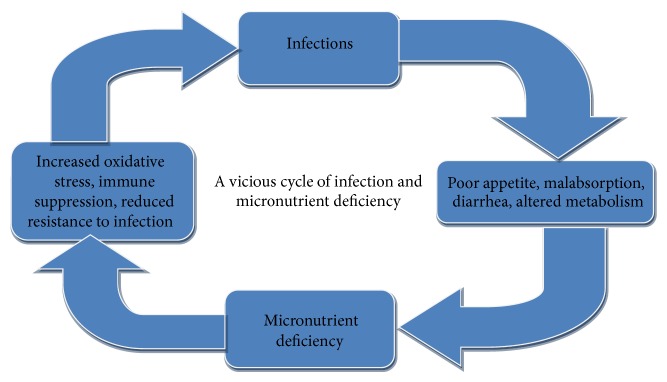
A vicious cycle of infection and micronutrient deficiencies.

Malnutrition can lead to expression of overt disease among individuals with latent infection by weakening the immune system. Malnutrition can make a person more susceptible to infectious diseases, and infection also contributes to malnutrition (Figure 1). An inadequate dietary intake results in stunting, lowered immunity, mucosal damage, invasion by pathogens, and impaired growth and development in children [27–29]. The interaction between micronutrient deficiency, infection, and immunity has been well documented. Infection may lead to micronutrient deficiencies and micronutrient deficiencies may affect the risk of infectious disease morbidity [22, 27–29, 45, 51, 52], which causes a vicious cycle. As seen from the conceptual framework presented in Figure 1, the effects of an infection are mediated via the acute phase response and localized lesions, leading to reduced intake and absorption which results in an increased utilization and loss of micronutrients. A micronutrient deficiency may affect the risk of infection with a specific infectious agent as well as the severity of the infectious disease morbidity. These effects are mediated through pathogenicity of the infectious agent and hosts immunity [53].

## 5. The Influence of Micronutrient Deficiency on the Progression/Mother to Child Transmission and Treatment Outcomes of HIV/AIDS

### 5.1. Micronutrients on the Progression of HIV/AIDS

The progression time of HIV infection to AIDS and from AIDS to death is of highly variable length. The examinations on the relationship between micronutrient deficiencies and HIV disease progression began in 1990s [54]. An inverse correlation between serum selenium concentrations and HIV disease progression including CD4 cell counts, opportunistic infections, and viral load has been reported by numerous authors [55, 56]. Low plasma or serum selenium concentrations were reported among symptomatic HIV patients as compared to symptom-free HIV-positive subjects [55]. Similarly, lower serum levels of selenium were reported in patients with a CD4 count less than 400 cells/mm^3^ of blood [57]. Another study reported that the occurrence of opportunistic infections was more frequent among patients with lower serum selenium concentration [17, 19, 58]. Moreover, it has been reported that mean serum selenium levels were significantly lower in patients at CDC HIV stage B and C as compared to healthy subjects and to HIV stage I patients [59]. In one study, low serum selenium levels increased the risk of HIV-related mortality by more than ten times [60]. Likewise, vitamin A status as an important cofactor in HIV progression has been reported. Low vitamin A concentrations were significantly associated with CD4 T-cell counts and increased progression to AIDS and as a result increased risk of mortality in HIV infected people [61, 62]. In Ethiopia, vitamin A deficiency has been reported as a severe public health problem among HIV infected patients [20, 21]. Low serum zinc levels in HIV patients are also reported in Ethiopia [17, 18, 63].

### 5.2. Micronutrients Deficiency on Mother to Child Transmission and Pregnancy Outcome

In Sub-Saharan countries, only 50% of women living with HIV were receiving antiretroviral medicine for PMTCT in the year 2010 [64]. Transmission of HIV from mother to infant can occur in utero, during delivery, or through breastfeeding [65]. Vertical transmission rates of HIV without any preventive measures are estimated to be 25–35% in developing countries [66]. Both maternal and child factors affect vertical transmission, and many of these factors relate to nutritional status. There has been concern of increased risk of HIV transmission from mother to child, with particular micronutrient deficiencies.

Vitamin A deficiency which is high among Ethiopian HIV-positive pregnant women [20] was first correlated with increased vertical transmission of HIV in Africa [67]. This has implications for potential clinical importance particularly in African regions where accesses to other forms of treatment are virtually impossible. Observational studies in sub-Saharan Africa have shown significantly increased rates of mother to child transmission of HIV [67, 68] and infant mortality [68, 69] among HIV-infected women with low serum vitamin A levels.

On the other hand, a study in African women showed that vitamin A supplementation was not associated with decreased HIV transmission [70]. However, it had positive effects on pregnancy outcomes such as decreasing preterm births, lowering the transmission rate in preterm babies, and reducing the incidence of low birth weight deliveries [70]. In addition, a study in Tanzania on pregnancy outcomes found that multivitamins decreased the risk of low birth weight, severe preterm birth, and fetal death while increasing CD4, CD8, and CD3 lymphocytes [71]. These results has important public health implications because preterm delivery rates of HIV-1 infected mothers can reach up to 42% in African countries and are associated with increased mortality and morbidity [71].

### 5.3. Micronutrients Deficiency and Oxidative Stress during HAART

HIV infection is accompanied by severe metabolic and immune dysfunctions. Oxidative stress is one of the dysfunctions which results from the imbalance between reactive oxygen species (ROS) production and antioxidants concentration [72]. Exposure to oxidants challenges cellular systems and their responses may create conditions that are favorable for the replication of HIV which is an increasing cause of morbidity and mortality among HIV/AIDS patients [73]. Currently, however, introduction of HAART has led to a decrement of viral load and a quantitative and qualitative improvement of the immune functions in patients, especially CD4+ count. This results in a decrement of infectious complications and global clinical improvement [74, 75]. But HAART also plays a role in oxidative damage to DNA and membrane polyunsaturated fatty acids, which later on generates more free radicals potentiating the cellular damage [76]. Therefore, an HIV infected individual on antiretroviral therapy is exposed to two courses of free radical injury: one is from the virus itself and the other from the antiretroviral drugs. Hence, in areas where multiple antioxidant micronutrient deficiency is common, an increased oxidative stress is expected among those on HAART. However, it remains to be determined whether multiple antioxidant micronutrient supplementations will have any effect on oxidative stress or viral replication and disease progression.

## 6. The Influence of Micronutrient Deficiency on the Transmission, Drug Resistance Development, and Treatment Outcome of TB

Malnutrition is more common in patients with active tuberculosis than in people without TB [77]. Weight loss, including loss of lean body mass, is a well-recognized symptom of the disease. A study conducted in Ethiopian TB patients showed that low body mass index (BMI < 18.5 kg/m^2^) was common and it was observed among 65.4% of TB patients and 71.6% of TB/HIV coinfected patients. Severe malnutrition (BMI < 165 kg/m^2^) was observed to be more common among TB/HIV coinfected patients [17]. Although generalized malnutrition has been commonly described during active TB, less is known about micronutrient status and TB disease pathogenesis [78]. However, the concentration of vitamins, minerals, and trace elements all have key roles in metabolic pathways, cellular function, and defense against TB [32, 79].

In the era before the introduction of TB chemotherapy, vitamin D rich cod liver oil and exposure to sunlight were once a part of regular therapy for TB [80]. Vitamin D plays a role in macrophage activation and was shown to be a key factor in host resistance to tuberculosis [81]. In addition, vitamin D downregulates the transcription of virulence factors that are important for the intracellular survival of* M. tb* in macrophages [82, 83].

Susceptibility to* M. tb* infection and progression to active TB may be increased by vitamin D deficiency [82, 84]. Abnormalities in vitamin D status are influenced by dietary, genetic, and exposure to sunlight. In addition, genetic variations in vitamin D receptor were identified as a major determinant of the risk for TB among Africans [85].

In Ethiopia, in spite of abundant availability of UV radiation, it has been reported that the population from Addis Ababa situated in tropics had a high rate of biochemical vitamin D deficiency [86]. Increased risk of vitamin D deficiency in darker skinned individuals is due in part to decreased dermal synthesis of vitamin D as a result of the absorption of UV radiation by the increased melanin pigmentation [87]. Vitamin D deficiency helps the disease to progress rapidly to the active form.

In recent years, rates of drug-resistant TB have been spreading fast across the world, causing alarm among public health officials and prompting calls for more research into new and more effective treatments. The emergence of multidrug-resistant TB (MDR-TB), where the bacteria are resistant to both rifampicin and isoniazid, extensively drug-resistant (XDR-TB), where the bacillus is additionally resistant to fluoroquinolones and at least to one injectable agent (such as amikacin, capreomycin, or kanamycin), and the more recent form which is resistant to all anti-TB drugs represents an emerging problem in the struggle to contain TB [88–90].

Vitamin A is an important immune enhancer that has been shown to increase lymphocyte proliferation in response to antigens and to potentiate antibody responses to T-cell-dependent antigens and inhibit apoptosis. Vitamin A is also important in maintaining the integrity of epithelial surfaces. Deficiency in vitamin A leads to reduced levels of secretory immunoglobulin A in mucous and, therefore, to a weakening of the local barriers to infection [91–93]. However, in Ethiopia, vitamin A deficiency among TB patients is extremely high, occurring in about 60% of patients with TB [16, 94].

Numerous studies have reported decreased antioxidants levels, disturbed glutathione metabolism, and enhanced spontaneous generations of reactive oxygen species (ROS) in TB patients [95, 96]. For that reason, an increased level of ROS is the main factor to lower concentrations of antioxidants in TB patients. To make matters worse, inadequate dietary intakes of antioxidant compounds that are capable of reacting with and inactivating ROS result in further ROS generation which leads to an increased utilization of endogenous antioxidants. Therefore, these oxidant-antioxidant imbalances (oxidative stress) may represent a pathogenic loop that results in markedly enhanced oxidative stress during TB infection [97, 98].

In Ethiopia, it was reported that levels of the antioxidant vitamins C, E, and A were considerably lower in TB patients than in healthy controls; particularly high concentrations of lipid peroxidation products were seen among those who were coinfected with HIV [94]. In another study conducted in northwest Ethiopia, low concentrations of trace elements such as zinc, iron and selenium were also reported [17]. Whether single or multiple antioxidant supplementations will improve TB treatment outcome or are of importance for its prevention requires in depth future prospective studies.

## 7. The Relationship between Intestinal Parasitic Infections and Micronutrient Deficiency

Malnutrition and intestinal parasitic infections are common public health problems in developing countries. Malnutrition and parasitic diseases have a strikingly similar geographical distribution with the same people experiencing both diseases together for much of their lives [94]. In Ethiopia, intestinal parasitic infection and malnutrition still constitute a major health challenge with the resultant clinical and social impact on the people [99–102].

As micronutrient deficiencies disrupt the function of various immune system components that increase vulnerability to various infectious diseases [24–29], intestinal parasitic infections affect the micronutrient status by decreasing nutrient intake due to loss of appetite, decreased nutrient absorption as a result of intestinal damage and malabsorption, and nutrient losses arising from diarrhea and increased urinary excretion [18, 19, 45–49]. The consequences of such coexistence deleteriously affect the immune mechanisms of the host [103].

Basically, immune responses to infectious agents engage two antagonistic, reciprocally cross-regulated classes of T helper cells: type-1 and type-2 T helper cells. Type-1 T helper immune cells are responsible for cell-mediated immunity against bacterial, protozoal, viral, and intracellular parasitic infections whereas type-2 T helper cells mediate antibody-dependent immunity against extracellular parasites including intestinal helminthes [104].

Intestinal helminth infection leads to micronutrient deficiency [18, 19, 45–49]. In turn, micronutrient deficiency decreases immunological response against intestinal helminthes [24–29]. Evidence suggests that type-2 immune response may play a crucial role in reducing the severity of acute disease upon helminth infection [105] resulting in chronic helminth infection. In this case, type-2 T helper cells produces a dominant pattern of cytokine immune effectors capable of downregulating type-1 T helper cells response [14, 104, 106–109], increasing vulnerability to other intracellular infections like HIV and TB [14, 108, 109].

Other studies proposed that undernutrition may prevent the expression of the dominant type-2 phenotypes and that energy deficiency [110], vitamin A deficiency [111], and protein deficiency [110] cause overexpression of type-1 T helper cells cytokine IFN-*γ* and consequently downregulation of essential type-2 T helper cell cytokines. The absence of type-2 T helper cell cytokines and their effectors results in prolonged survival of helminthes. In addition, current evidence shows that zinc deficiency is characterized by declines in several type-2 T helper immune effectors in mice [112]. In Ethiopia, several studies reported multiple micronutrient deficiencies in different segments of the population [16–19, 21, 66, 100, 107].

## 8. Conclusion 

From the extensive literature, it can be concluded that effect of single and multiple micronutrient deficiency on pathogenesis of HIV, TB, and intestinal parasitic infections is of immense clinical and public health importance in Ethiopia where these diseases often coexist. Furthermore, the bidirectional interactions between multiple micronutrient deficiencies and infectious diseases may have potentially enormous long term developmental and societal impacts in the country. Therefore, it is needless to point out that coinfection with two or more pathogens may even make the problem worse. Thus, the authors hope that this information will fuel the development of new ideas and research studies focused on investigating the effect of single or multiple micronutrient supplementations on infection transmission, immune status, diseases progression, morbidity, mortality, and treatment/vaccine outcome. Further investigation is also needed to evaluate the prophylactic and therapeutic potential of micronutrient interventions in augmenting chemotherapy during coinfection.

## Figures and Tables

**Table 1:** Selected micronutrient levels in serum of blood donors (apparently healthy controls), pregnant women, and tuberculosis patients by HIV serostatus in Ethiopia.

Trace elements	Controls (blood donors) [16–20]		Pregnant women [20]		Tuberculosis patients [16, 17]		Diarrheic patients [18, 19, 21]	
	HIV− (*n* = 68)	HIV+ (*n* = 32)	HIV− (*n* = 327)	HIV+ (*n* = 42)	HIV− (*n* = 81)	HIV+ (*n* = 71)	HIV− (*n* = 97)	HIV+ (*n* = 109)
Mg (mg/dl)	2.85 ± 0.61^*^	—	2.43 ± 0.82	2.14 ± 0.86	—	—	1.76 ± 0.34	1.68 ± 0.26
Ca (mg/dl)	14.41 ± 3.61	11.11 ± 1.46	14.39 ± 4.69	13.41 ± 5.22	—	—	8.38 ± 1.97	7.82 ± 1.23
Fe (*μ*g/dl)	480.9 ± 449.0	288.3 ± 194.8	561.97 ± 415.23	485.86 ± 275.23	280.82 ± 314.31	265.99 ± 369.91	352.06 ± 351.23	420.82 ± 665.14
Cu (*μ*g/dl)	140.3 ± 47.95	166.2 ± 45.4	240.19 ± 73.55	239.59 ± 81.47	188.19 ± 58.65	176.59 ± 63.19	113.51 ± 38.28	126.83 ± 34.91
Zn (*μ*g/dl)	88.1 ± 4.02	77.2 ± 25.3	75.19 ± 44.79	76.30 ± 125.43	81.14 ± 14.16	73.65 ± 37.66	62.39 ± 43.64	68.13 ± 44.53
Se (*μ*g/dl)	9.6 ± 4.37	10.2 ± 4.5	10.49 ± 4.24	8.0 ± 4.71	8.86 ± 3.93	7.55 ± 2.63	6.99 ± 4.26	5.90 ± 2.79
Vitamin A (*μ*g/dl)	42.83 ± 20.37	25.83 ± 14.28	31.57 ± 12.79	27.56 ± 12.01	21.57 ± 13.81	19.98 ± 13.28	24.18 ± 15.68	23.57 ± 16.77
	*N* = 92	*N* = 30	*N* = 379	*N* = 44	*N* = 107	*N* = 115	*N* = 101	*N* = 110

^*^Mean ± standard deviation.

Trace elements were measured by ICP-MS (inductively coupled plasma mass spectroscopy) and vitamin A was measured by HPLC (high performance liquid chromatography).

## Abbreviations

HIV: Human immunodeficiency virus
TB: Tuberculosis
WHO: World Health Organization
AIDS: Acquired immunodeficiency syndrome
HAART: Highly active antiretroviral therapy
BMI: Body mass index
MDR-TB: Multidrug-resistant tuberculosis
XDR-TB: Extensively drug-resistant tuberculosis
ROS: Reactive oxygen species.
